# Coexisting with wildfire: strengthening collective capacity by changing the status quo

**DOI:** 10.1186/s42408-024-00290-y

**Published:** 2024-07-22

**Authors:** Christine Eriksen

**Affiliations:** Institute of Geography, University of Bern, Hallerstrasse 12, 3012 Bern, Switzerland

**Keywords:** Climate change, Coping capacity, Diversity, Equity, Inclusivity, Privilege, Social science, Structural inequality, Wildfire, Workforce sustainability

## Abstract

This article is the fuller written version of the invited closing plenary given by the author at the *10th International Fire Ecology and Management Congress*. The article provides a consideration of our capacity to cope, care, and coexist in a fiery world from a social and structural point of view. It focuses on privilege as the root cause of a long and troublesome history within the wildfire profession of not valuing all generational knowledge equally, not treating all cultures with the same respect, not embracing diversity and inclusion, and not affording the same status to all disciplines and voices. The article argues that we can strengthen our collective capacity to coexist with wildfire by embracing local and indigenous fire stewardship practices, by enabling workforce diversity and inclusive leadership culture, and by providing sustainable working conditions for wildland firefighters. To do so requires individual and collective noticing of what is wrong, and everyday action steps towards equity.

## Resumen

Este artículo es la versión escrita completa de la disertación brindada por la autora en el cierre del plenario del 10mo. Congreso Internacional de Ecología y Manejo del Fuego. El artículo expone una consideración sobre nuestra capacidad de ajustarnos, cuidar, y coexistir en un mundo exaltado desde el punto de vista social y estructural. Se enfoca en los privilegios como la causa radical de una larga y tormentosa historia dentro de la profesión y de no evaluar todos los conocimientos generacionales de la misma manera, el no tratar las diferentes culturas con el mismo respeto, no abrazar la diversidad e inclusión, y no considerar el mismo estatus para todas las disciplinas y voces. El artículo argumenta que podemos reforzar nuestra capacidad colectiva para coexistir con los fuegos de vegetación mediante la inclusión de moradores locales y gente indígena en relación a sus prácticas de manejo del fuego, mediante la diversidad en nuestra fuerza de trabajo e inclusión de los liderazgos culturales, y mediante la provisión de condiciones de trabajo sostenibles en el tiempo para los brigadistas de incendios. Para ello se requiere el anoticiarse de manera particular y colectiva de lo que está mal y de las acciones diarias que tiendan hacia la igualdad.

## Introduction

This article is the fuller written version of the invited closing plenary given by the author at the *10th International Fire Ecology and Management Congress* in Monterey, California, on 7 December 2023 (AFE 2023). The article provides a consideration of our individual and collective capacity to cope, care, and coexist in a fiery world from a social and structural point of view. To do so, the article is framed by two familiar concepts in wildfire management: anchor points and firelines. Wildland firefighters set up an anchor point to gain an advantageous location from which to start constructing a fireline, with the hope to create a burn boundary (USFS n.d.). In this way, both notions hold deep symbolic meaning, as they play a crucial role in controlling the spread of wildfire while upholding the safety of firefighters (Desmond 2007; Santos 2016; Thomas 2022).

Yet, notions of anchor points and firelines can also act as poignant metaphors in acknowledging our growing inability to manage wildfire in the Anthropocene (Eriksen 2023). While climate change is often depicted in public debates as being the main culprit of our fiery crisis, it is an established fact that social factors are equally at play. It is a result of the relationship between land use changes, fire exclusion, tree mortality, and a hotter and drier climate, which acts as a positive feedback loop that is feeding catastrophic wildfires (Bowman et al. 2020). Human activity has altered the parameters of Earth systems, making firelines less effective in controlling the known and emerging consequences of climate change, including wildfires (Petryna 2018; Eriksen and Ballard 2020). Today, environmental management can feel akin to fighting fires of such escalating intensity that neither an anchor point can be found, nor a fireline established (Celermajer 2021; see also Eriksen 2022a).

Our transformation of Earth systems necessitates a transformation of the relationship between people and fire. The need for this transformation has been a long time in the making (Pyne 2022). Yet, rather than face the fire, so to speak, society at large has mostly chosen to kick the can down the road, in a manner akin to Vaillant’s description below of how climate scientists have been ignored despite the long-standing and compelling evidence linking fossil fuels and CO_2_ emissions with global warming:

What the atmosphere and oceans are telling us is that carbon dioxide doesn’t get the respect it deserves. Others have been saying this, too – for a long time. … Until very recently, most of them have been tuned out, brushed off, or appeased in ways that bear a strong resemblance to the experience of people reporting incidents of sexism or racism: “Where? I can’t see it.” (Vaillant 2023: 297;)

Vaillant’s reference to the prevalence of willful blindness towards sexism and racism is a throwaway line, which he does not elaborate on further in his book. Yet, the point he makes is, in my opinion, worthy of further reflection, as it points to a critical problem: namely, the way we—as individuals and as a society—tend to willfully ignore issues that undermine essential progress. While I share the aspirations of the *10th International Fire Ecology and Management Congress*’ theme of “Igniting Connection [by] celebrating our fire family across generations, cultures, and disciplines” (AFE 2023), twenty-odd years as a fire social scientist has instilled a strong belief in me that you cannot truly ignite connections to transform our relationship with fire without continually acknowledging how each of us are privileged or marginalized depending on our social characteristics, be that our age, gender, sexuality, race, ethnicity, disability, education, etc. Our so-called “fire family” has a history of not valuing all generational knowledge equally. It does not treat all cultures with the same respect, and it struggles to embrace diversity and inclusion. Moreover, different disciplines are not afforded the same status when it comes to the projects we fund, the insights we publish and reproduce, or the voices we choose to amplify (Eriksen 2022b). To grow stronger together, to strengthen our collective capacity to coexist with wildfire, we must fully address the injustices of the past that continue to haunt us today.

“A recovery can be in resistance” (Ahmed 2021: 287)—a resistance to forget, a commitment to learn from our mistakes. Change for good requires momentum, and such momentum is usually driven by sustained and painstakingly hard work. Change takes time because it takes time to build social momentum. As Solnit (2009: 119) highlights, “Disaster shocks us out of slumber, but only skilful effort keeps us awake.” When it comes to the relationship between people and wildfire, such skillful effort has been increasing since the 2009 Black Saturday bushfires in Australia (e.g., McLennan and Handmer 2012; Christianson 2014; Moritz et al. 2014; Eriksen and Simon 2017; de Vet et al. 2019; Billings et al. 2021; Nolan et al. 2021; Schinko et al. 2023). The frequency, intensity, scale, and impact of the Black Saturday bushfires (Teague et al. 2010), and a disproportionate number of other wildfires around the world since then, has stripped away the surface of social structure and revealed a grim picture of long-standing social and structural inequality.

It is now an unquestionable fact that socially diverse people can experience exposure, vulnerability, and adaptive capacity quite differently based on historical and structural inequities (Eriksen et al. 2020; Simon and Eriksen 2021). This fact has added urgency to a vital question: How can we co-exist with fire in a hotter and drier climate? I believe we already know the answers to this question. There are different answers for different parts of society, of course—insights that are beyond the scope of this article. Yet, common to all these issues is the struggle to implement the changes that are needed, partly due to the lifestyle change this would require for many, but in large part also because of ingrained historical and structural inequities (e.g., Ladino et al. 2022). In the remainder of this article, my focus specifically concerns the urgent need to transform the unsustainable social and structural issues that impact the workforce with which we attempt to manage wildfire.

## Transforming wildfire management: a spotlight on social and structural issues

Three particular social and structural issues underpin many of our current struggles in wildfire management: (1) a long and troublesome history of marginalizing Indigenous people and their fire stewardship practices; (2) a toxic patriarchal workplace culture that discriminates against women, BIPOC,^1^ and LGBITQ + 2 people; and (3) an exodus of wildland firefighters from the workforce due to longer and harder fire seasons that make the workload, poor pay, and lack of social benefits unsustainable. While it is wildfire itself that has made the need for change so blatantly obvious, it is often the people whose lives and voices have been systematically marginalized or discriminated against over time that have driven the momentum to change these unsustainable social and structural issues.

### Indigenous ecocultural burning

For Indigenous people, this particular point in time—the fiery crisis we now face—is by no means the first time they have had to cope with devastating change. Ecocultural fire is such an apt expression for indigenous burning practices because it highlights how indigenous fire stewardship has been tried and tested both ecologically by climatic and environmental change for millennia, and tried and tested culturally for centuries, as Indigenous people have been persecuted, policed, or denied access to the land, resources, ancestral ties, and sovereignty that underpin their ecocultural knowledge and practices (Langton 1998; Stewart et al. 2002; Anderson 2006; Gammage 2011).

Given these long histories, it is quite extraordinary that when I first visited California as a researcher in 2011 and started collaborating with Don L. Hankins at California State University, Chico to give voice to Indigenous fire knowledge keepers in southeast Australia and California, the dominant narrative among fire practitioners and fire researchers was still a dismissal of indigenous fire stewardship as being either a thing of the past or as being incompatible with the land management practices that govern a demographically altered world (Eriksen and Hankins 2014, 2015; Eriksen 2022c). As one Indigenous fire steward shared with us, it all comes down to the ability to nurture long-term ecocultural thinking—to connect the past with the present and the future:

Traditional Knowledge Revival Pathways (TKRP) – I think the ‘R’ that’s in that acronym, you know ‘revival’, is really important, because when you try and explain this to a white audience, and particularly the white fire management, they want to see it. “Well, where is it? You show me this special formula of indigenous knowledge that’s going to solve everything. Go on, show us now!” It’s about reviving. It’s about bringing that knowledge back and they’re like “We don’t have the money and time. We need real solutions now.” They’re not long-term thinkers. (Indigenous national park ranger, NSW, June 2011)

In the dozen or so years that have passed since then, catastrophic wildfires have demonstrated the dire consequences of excluding ecocultural fire from the landscape. At the same time, landscapes managed with what is increasingly referred to as “good fire” have shown their ability to mitigate the spread of wildfire while sustaining a healthy ecosystem (e.g., Kristoff and Christianson 2023; Maclean et al. 2023; Stephens et al. 2023; Wu et al. 2023; Eisenberg et al. 2024). One of the silver linings of catastrophic wildfires is therefore the gradual opening it has provided to Indigenous fire stewards to tell their stories, to participate in fire management, policy, and legal debates, and to rejuvenate their ecocultural burning practices slowly but surely (e.g., Mistry et al. 2016; Steffensen 2020; Williamson et al. 2020; Hankins 2021; Long et al. 2021; Smith et al. 2021; Williamson and Weir 2021; Hoffman et al. 2022; Muskrats to Moose Project 2023; Rodríguez et al. 2023; Weir 2023).

### Gender diversity and inclusive leadership

Another silver lining of catastrophic wildfires is how the need for a bigger and stronger wildland firefighting workforce has facilitated a growing recognition of not just the need for a more diverse workforce but also the advantages of a more inclusive leadership and workplace culture. Women, let alone BIPOC and LGBTIQ + people, were still an underrepresented and, by many, also an unwanted part of the workforce when Pacholok (2013; see also Pacholok 2009) and I (Eriksen 2014) published our respective books on gendered dimensions of wildfire in mid- and late-2013. Our research made visible what until then had predominately been a hushed internal matter about unsustainable working cultures, or, at times, a legal struggle for equal rights in the workplace, as portrayed in the seminal work by Enarson (1984; see also Enarson and Morrow 1998; Enarson and Pease 2016). It was also the beginning of a more systematic examination by researchers, journalists, and organizations alike of a toxic workplace culture that harbor a distressing amount of gender discrimination and sexual harassment (e.g., Ainsworth et al. 2014; Eriksen and Waitt 2016; Eriksen et al. 2016; Reimer and Eriksen 2018, 2022; Eriksen 2019; McQuerry et al. 2023; Ragland et al. 2023).

In the years after our books were published, many attention-grabbing headlines made the news about the unsafe working conditions for female firefighters (e.g., Langlois 2014, 2016; Fears 2016; Gilpin 2016a, 2016b, 2021; Joyce 2016, 2018), and the Association for Fire Ecology (AFE 2016) appointed a committee, which I was a part of, to gather evidence with which to take a stand against harassment and discrimination. The momentum is growing. Today, concerted effort is being made by many to attract and retain women in the profession through training, networking, and policies that strive towards equal opportunities, informed leadership, and a workplace culture where toxic masculinity is challenged rather than celebrated (e.g., Wallien 2017; Rom 2018; Aldrich 2019; Potter 2020; Kutz 2022; Girls on Fire Inc 2023; WTREX 2023; Nikirk 2024). At the time I was writing my book, women made up about 5% of wildland firefighters in North America. Today, that number has grown to about 12% in the USA (Fahy et al. 2022; USFA 2023).^3^ These statistics clearly show that, while improvements have been made, we still have a long way to go to achieve a truly gender diverse workforce.

### Healthy, safe, and sustainable working conditions

Structural and cultural workplace changes are challenging to implement at the best of times. The heavy toll longer fire seasons and more devastating wildfires are taking on the wildland firefighting workforce and resource availability makes a sustained commitment to, and investment in, healthier and safer working conditions even more challenging to execute. Better pay might help retain experienced firefighters and attract new recruits, but without accompanying programs, benefits, and support, such as paid rest periods, physical and mental health support, and an inclusive workplace culture, wildland firefighting is clearly not a sustainable profession in our fiery world (Reimer and Eriksen 2022; Davis et al. 2023; Granberg et al. 2023; Held et al. 2024).

These big consequential fires, when they happen, are going to be labelled as a natural disaster, or climate change, or a lack of forest management, depending on what side of the aisle you are on. It’s not going to be that we couldn’t hire enough people and that Congress didn’t pass enough legislation – but that’s what those of us doing the job are seeing. (Anonymous U.S. wildland firefighter, quoted in Canon 2023)

The upshot of unsustainable working conditions is not just too few hands on deck, along with the known risks and potentially life-threatening aspects of the job. Wildland firefighters are also experiencing increasing rates of depression, PTSD,^4^ alcohol and drug abuse, and death by suicide (AFE 2016; Singer 2021; Granberg et al. 2022; Cooper and Duncan 2023). For a workforce that is already struggling to deal with harassment and discrimination, it is also important to remember that, “Precarity can make it harder not only to make a complaint but to support a complaint, especially if the complaint is about the conduct of permanent or senior members of staff” (Ahmed 2021: 229).

## Understanding the role of privilege in the wildfire profession

As the above spotlight on social and structural issues shows, it is by now well understood that a thriving workforce rests on the need to recruit and retain people who are valued regardless of their social characteristics, such as race, ethnicity, gender, sexuality, and cultural upbringing. We know that a healthy and safe workplace rests on equitable working conditions that provide a decent salary, social benefits, and health care. We know that healthy environmental conditions rest on local and indigenous environmental knowledge that have been passed on for generations. It is also well established that integrated wildfire management depends on policies and governance that are driven and supported by in-depth insights from the social sciences (alongside forestry, fire ecology, etc.). Yet, instead of making the wholesale social and structural changes needed to enable all these factors, we tend to still put them in the “too hard” basket, willfully ignore them, or proactively fight to stop such change. Why? A short and simple answer to this question is privilege.

The concept of privilege refers to a special right, advantage, or immunity granted or available only to a particular person or group, usually because of their gender, sexuality, race, ethnicity, class, or education. In his examination of how to undo privilege, Pease (2021) highlights the often unchartered and latent terrain of how privilege is socially and structurally reproduced in everyday life. Pease believes that too much attention has been focused on the responsibility of those who are oppressed, and too little attention has been given to how those in privileged groups reproduce inequality. As he states:

Part of the problem is that it is very difficult to get the issue of privilege on the agenda because it is so well legitimated. Privilege is not recognised as such by many of those who have it. Privilege appears to be natural. Therefore, it is necessary to ‘unmask’ privilege and make it more visible so that its consequences can be addressed. (Pease 2010: ix)

At the same time, as many of those who have privilege do not recognize it as such, those who do not have it see and feel the consequences of disenfranchisement all the time. In her examination of complaint activism, Ahmed (2021: 164) unpacks “how the more we challenge structures, the more we come up against them.” She explains how:

… to keep information inside is how the house can appear in order from the outside. … Even when complaints are not about sexism and racism, sexism and racism come up in what comes down. Race and gender: they are always in there, in the situations we find ourselves in, for those of us who are not white, not men. (*ibid*: 253)

Ahmed continues:

You come to learn how violence against those who challenge violence is how structures are maintained. You come to realize that some are more readily targeted. … you come to witness the violence of the status quo when you challenge the violence of the status quo. (*ibid*: 283)

It is because of such systemic inequality, and the inability of some to recognize it, that generational knowledge, cultural diversity, and interdisciplinarity are a double-edged sword in the wildfire profession. While generational knowledge, cultural diversity, and interdisciplinarity provide the answers needed for vital change, history also shows how certain generations, certain cultures, and certain disciplines inherit taken-for-granted privileges that give them a vested interest in either maintaining the status quo or changing some things but not others.

Acknowledging this double-edged sword helps us see how generational knowledge can either refer to the tried and tested local and indigenous ways of knowing, which can be passed on to future generations so they can (re)learn how to coexist with wildfire; or it can refer to the legacy of historical and structural injustices, such as colonialism, racism, and patriarchy, that are built-in to our organizational structures, political agendas, and everyday cultural norms, where they act as barriers to change. Cultural diversity can either refer to a strengthening of our capacity to cope in a fiery word by evening the playing field for all people, regardless of gender, sexuality, ethnicity, race, and education; or it can be a buzzword used by institutions and in policies to appear inclusive while reproducing the status quo. Similarly, interdisciplinarity can either refer to true collaboration across disciplines and methodological approaches, which can facilitate new or improved ways of seeing and doing; or it can be a disguise used by scientists in dominant disciplines who include diverse collaborators, such as social scientists and Indigenous people, on grant proposals to make their proposals more competitive, but then systematically side-line or belittle these collaborators once the funds have been secured. In this way, “Diversity can be how you are colonized again; [how] what you have, what you are, is taken from you [again]” (Ahmed 2021: 247).

It is difficult to change such privilege because, until recently, many dominant groups have rarely been challenged to think about their own dominance, let alone made to understand how their dominance is based on unearned advantages, and why relinquishing these advantages will benefit all of society. It is also difficult because White, heterosexual, cis-gender men trained in science and engineering have dominated universities, wildland fire, and emergency management for so long that social norms are written into policies and operational guidelines precisely because they are mainly written by, and for, dominant groups. This approach is so blatantly inadequate today because more people, from more walks of life, live at risk from wildfire than ever before. But it is also wholly unsustainable because the White, gendered structures that are so deeply ingrained in the operational procedures and cultural norms of wildland fire are inhibiting the inclusion and retention of more women, BIPOC, and LGBTIQ + people into a dwindling workforce. It is also wholly incapable of supporting White, cis-gender men who struggle to fulfill unhealthy cultural demands for heroic forms of masculinity in the face of catastrophic wildfires.

## Undoing privilege: noticing and acting

Some of you, readers, know only too well how much more weight you must bear because of the lack of support you receive in raising the injustices you are experiencing—be it as a woman, BIPOC, or LBGTIQ + person, or as an overworked and underpaid wildland firefighter. If there is one thing catastrophic wildfires is teaching us, it is that we must learn from what we have failed to achieve. One of the ways that we can do this is by heeding the advice from activist research, which painstakingly shows us that when “weight is distributed more, each person carries less”, and how “when a complaint is shared, you can also widen the range of activities undertaken” (Ahmed 2021: 282).

It was our differences that gave us the vantage point to see, together, what any one of us could not fully see alone: to see the extent of the condition we were in. (Whitley et al. 2021: 264)

By diversifying our workforce and empowering leaders to support such diversity, we can transform privilege and inhibiting social norms (Reimer and Eriksen 2018, 2022; Pease 2021). Large-scale programmatic and policy changes and infrastructure investments, as well as larger forums and position statements by associations, like those mentioned above, play a critical role in calling out problematic dynamics and influencing political and institutional change. But to truly intervene in what is wrong also requires noticing it is wrong in an everyday context, and that we must all do regardless of where we work or live. As Ahmed argues (2021: 164; italics in original), “Noticing something can be what we do for others. … *to stop the same thing from happening*. “The same thing” could be thought of as an institutional legacy”—such as the institutional legacy in wildfire management that tolerates privilege and disregards individual and collective needs in favor of personal or political gains. By not noticing—due to ignorance or by turning a blind eye, this legacy ensures that the privileged are neither challenged nor enabled to change their discriminatory or marginalizing behavior.

**Fig. 1:**
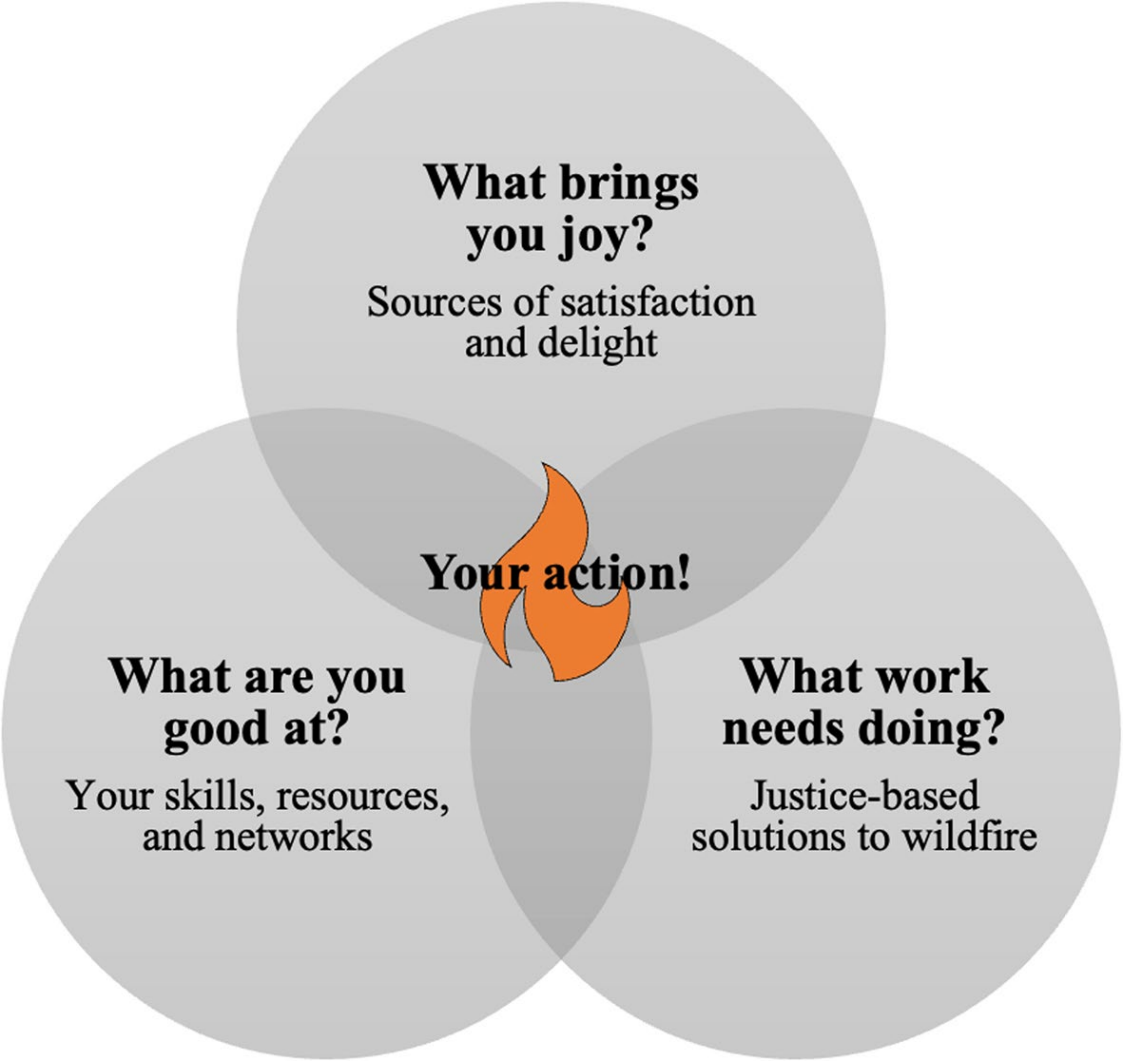
Leveraging your talents to create justice-based solutions for coexisting with wildfire (adapted from Johnson 2022)

Noticing is an important precursor for caring. Caring, in turn, provides incentive for proactively creating change in our everyday lives. This is not as daunting as it sounds! In her quest to facilitate just and joyful solutions for the climate crisis, Johnson (2022; see also Johnson and Wilkinson 2020) emphasizes that most of us are likely most powerful in our existing roles, where we have specialized knowledge and robust networks. She argues that if we identify what we are good at, what work needs doing, and what brings us joy, then we should actively seek to act where our individual answers intersect in a Venn diagram (Fig. 1).

Each step matters, no matter how big or small our everyday actions are, because we need justice-based solutions for change in every sector of the wildfire profession and in every at-risk community. One such step can be to support others to use their talents because, as Johnson (2020) stresses, this helps demolish societal barriers, like racism and sexism, that prevent people from fully devoting themselves to findings and implementing solutions. In this way, we, as individuals, can strengthen our collective capacity as a society to coexist with wildfire.

If each and every one of us intervene in what is wrong by noticing it is wrong, and all of us take steps in our everyday lives to create an even playing field by doing what we are good at, and supporting others to do the same, we will start to find anchor points that will enable us to cope with the heavy toll catastrophic wildfires is taking on our physical and mental wellbeing—be it as firefighters, cultural knowledge holders, land stewards, wildfire survivors, residents at-risk, or as researchers. We can find inspiration and hope by supporting innovative initiatives by BIPOC and diverse young generations, such as my AFE (2023) co-presenters Royal Ramey from the Forestry and Fire Recruitment Program (FFRP 2023) and Ryan Reed and Kyle Trefny from the FireGeneration Collaborative (FireGen 2023), who, despite the social and structural inequities they are up against, have the courage to challenge the status quo. All these steps will help us undo privilege, so generational knowledge, cultural diversity, and interdisciplinarity can help us survive and thrive in a fiery world.

## Data Availability

Not applicable.

## Footnotes

1 Black, Indigenous, and People of Color (BIPOC).

2 Lesbian, Gay, Bisexual, Transgender, Intersex, Queer, and others (LGBTIQ +).

3 Obtaining gender disaggregated data for firefighters, which also distinguishes between wildland, volunteer fire service, and structural (career) firefighters is difficult in most countries. I could not believe my eyes when I came across this neat breakdown on the USFA (2023) website: in total, 9% of US firefighters are women; 5% of career fighters, 11% of the volunteer fire service, and 12% of federal wildland firefighters. Such disaggregated data should be the gold standard everywhere.

4 Post-traumatic stress disorder (PTSD).
